# Development of a Novel Tabletop Device With Suction and Sanitization of Droplets against COVID-19

**DOI:** 10.7759/cureus.34287

**Published:** 2023-01-27

**Authors:** Katsuya Okuhata, Mitsugu Fujita, Kenji Nakamura, Yuya Yanagi, Yusuke Sakai, Kazuki Kubo, Hiroyuki Kosaka, Hajime Monzen

**Affiliations:** 1 Department of Radiology, Kansai Electric Power Hospital, Osaka, JPN; 2 Center for Medical Education and Clinical Training, Faculty of Medicine, Kindai University, Osaka, JPN; 3 Department of Medical Physics, Graduate School of Medical Sciences, Kindai University, Osaka, JPN

**Keywords:** covid-19, portable device, sanitization, suction, droplet, aerosol

## Abstract

Background

Coronavirus disease 2019 and other viruses are transmissible by aerosols and droplets from infected persons. This study aimed to develop a portable device that can trap droplets and deactivate viruses, and verify whether the device in an enclosed room can suction droplets and sanitize them using a filter and an ultraviolet-C (UVC) light-emitting diode.

Materials and methods

The portable device was evaluated by placing it 50 cm away from the droplet initiation point. A particle image velocimetry laser dispersed into a sheet form was used to visualize the droplets splashed on the irradiated sagittal plane and captured using a charge-coupled device camera at 60 frames per second. The images were overlaid and calculated to determine the percentage of the droplets beyond the portable device. Droplets with a particle size larger than 50 µm that dispersed and were deposited more than 100 cm away were measured using a water-sensitive paper. The effect of UVC sanitization on viruses captured by a high-efficiency particulate air (HEPA) filter was determined using a plaque assay.

Results

The percentage of droplets was 13.4% and 1.1% with the portable device OFF and ON, respectively, indicating a 91.8% reduction. The deposited droplets were 86 pixels and 26 pixels with the portable device OFF and ON, respectively, indicating a 68.7% reduction. The UVC deactivated more than 99% of the viruses on the HEPA filter surface in 5 minutes.

Conclusions

Our novel portable device can suck and fall the dispersed droplets, and an active virus was not observed on the exhaust side.

## Introduction

Coronavirus disease 2019 (COVID-19) has spread worldwide, with droplets and aerosols from infected persons being the main source of transmission [1]. Air dilution through ventilation is recommended to reduce aerosol concentrations [2,3]. In an enclosed space, a large portable air purifier with a high-efficiency particulate air (HEPA) filter and clean air delivery rate (CADR) of 1200 m^3^/h can reduce aerosol concentrations to comparable or better than natural ventilation [4]. The aerosol collection rate depends on the CADR of the air purifier as well as the environment, such as the location of the people and the air purifier [5]. Therefore, the air purifier should be selected according to the environment where it will be used.

COVID-19 regulations are being relaxed according to national and regional rules, considering vaccination rates and hospital bed occupancy. Additionally, restaurants are reopening with limited capacity, and there are often opportunities to take off the mask in a crowded restaurant. Droplets from a cough are dispersed more than 200 cm, which exceeds the social distancing standard in Japan of 180 cm [6]. Therefore, it is necessary to develop preventions for droplet infection besides wearing a mask. It was recently reported that airflow toward the inlet port of a portable air purifier placed on a desk removes aerosols created by an opposing person by more than 60% [7]. Alternatively, clean air from the outlet port of the air purifier toward the ceiling diffuses aerosols into the room [8]. With regard to droplets created during dental treatment, there are suction devices that remove droplets in the oral or extraoral cavity, which are effective in preventing the spread of droplets and aerosols into the room [9]. Generally, the suction devices placed into oral and extraoral cavities are used near the area where droplets are generated. Therefore, it is unclear whether the air purifier placed on the table can suction cough droplets created by opposing persons using the airflow toward the inlet port.

The purpose of this study was to develop a portable device that can trap droplets and deactivate viruses, and to verify whether the device in an enclosed room can suction droplets created by an opposing person using the airflow and sanitize the droplets using a filter and ultraviolet-C (UVC) light-emitting diode (LED).

## Materials and methods

Characteristics of the portable device

**Figure 1:**
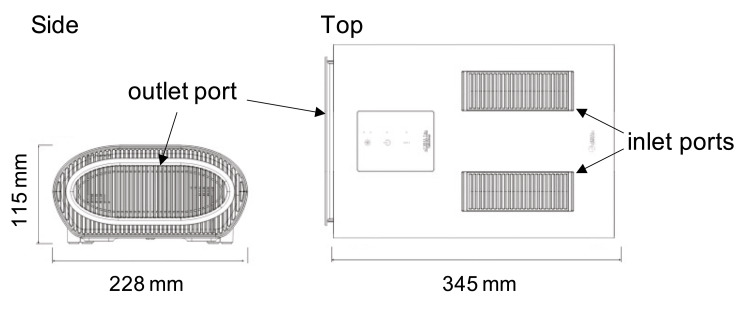
The developed portable device.

Figure 1 shows our developed portable device (eLENA Lin, Fujidenolo Co. Ltd., Aichi, Japan) that suctions air from its top and exhausts clean air from its side at a maximum CADR of 0.9 m^3^/min with low noise (59 decibels). There is a 150 mm (wide) × 150 mm (length) × 40 mm (height) HEPA filter and a UVC LED with a wavelength of 265 nm for the capture and deactivation of viruses, respectively. The HEPA filter has a collection efficiency atmospheric dust with a size of 0.3-0.5 µm from 99.865% to 99.984% at an airflow rate of 0.5 m^3^/min and 99.477% to 99.822% at an airflow rate of 1.0 m^3^/min. The HEPA filter can be replaced periodically to maintain suction power. The size of the portable device was 228 mm (wide) × 345 mm (length) × 115 mm (height) and its weight was 1.84 kg. The device was designed to have a low height when placed on a tabletop to not hide a person’s face or block views.

Percentage of suspended aerosols and vector analysis

**Figure 2:**
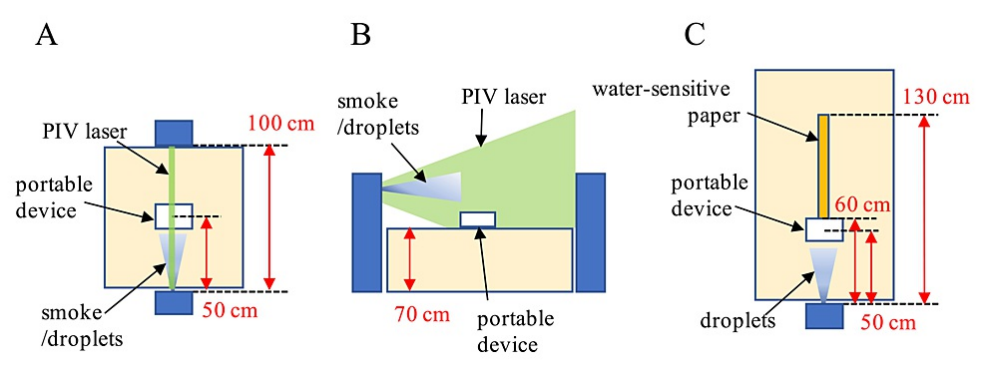
Experimental setup for droplet and aerosol visualization. (A) Dispersal point of the droplets or smoke, and the placement of the portable device. (B) Tracking the droplets and smoke for visualization on a two-dimensional optical pass at the sagittal plane of the particle image velocimetry (PIV) laser, (C) and visualization of deposited droplets on water-sensitive paper.

The portable device was placed on a table 50 cm away from a person to simulate two people facing each other across a 100 cm table (Figure 2A). A particle image velocimetry (PIV) laser (wavelength of 532 nm) with a laser pointer spread into a sheet was irradiated between the two people (Figure 2B). One person had smoke in their mouth and exhaled toward the person on the opposite side. The aerosols in the exhaled air were visualized by tracking the smoke suspended in the two-dimensional optical path of the PIV laser. The aerosols were captured using a charge-coupled device (CCD) camera at 60 frames per second. Each frame was overlaid to create a single image of the aerosol trajectory, and the region of interest (ROI) was set beyond the portable device. Aerosols in the ROI were binarized, and the percentage of aerosols normalized by the ROI area was compared when the portable device was OFF and ON. In addition, analysis software (Flow Expert 2D2C; Kato Koken, Kanagawa, Japan) was used to determine the mean value of the aerosol displacement vector.

Suction test of the droplets

As shown in Figures 2A, 2B, water droplets (droplets) created using a mist sprayer were directed toward the opposite person to simulate coughing, and the droplets were visualized on the two-dimensional optical path of the PIV laser. The droplets were captured at 60 frames per second using a CCD camera. Each frame was overlaid to create a single image of the droplet trajectory, and the ROI was set beyond the portable device. The droplets in the ROI were binarized, and the percentage of droplets normalized by the ROI area was compared when the portable device was OFF and ON.

A water-sensitive paper that turns blue when droplets larger than 50 µm adhere to it was placed beyond the portable device (Figure 2C). The droplets were sprayed 50 times with a mist sprayer because the droplets also evaporate in the air without deposition. To measure the number of deposited droplets, water-sensitive papers were digitized and binarized. The number of pixels which deposited droplets from the binarized image was compared when the portable device was OFF and ON.

Verification of the virus removal performance

An influenza virus was used to verify the performance of virus removal using the plaque assay method [10]. First, a human influenza A virus solution (1 × 10^6^ plaque forming unit (PFU) in one ml saline) was applied to 5 × 5 cm^2^ filter paper, which was then placed on the UVC LED side (inlet side) of the device. Filter paper soaked in saline solution was placed on the opposite side of the HEPA filter (outlet side). The portable device was run with the UVC LED ON and OFF for 5, 30, and 120 minutes, and then the filter papers were retrieved. The filter paper was put into a 50 ml conical tube and 4 ml of saline was added. The solution was centrifuged at 3,000 rotations per minute for 10 minutes, and the supernatant was collected. The Madin-Darby canine kidney cell line was then used to perform a viral plaque assay as described above. Results were obtained as PFU.

## Results

Percentage of suspended aerosols and vector analysis

**Figure 3:**
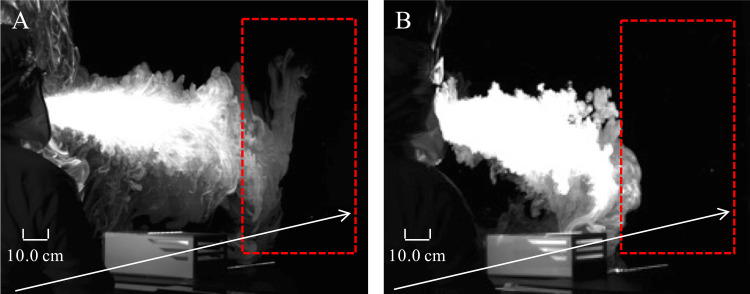
Analysis of the percentage of suspended aerosols when the portable device was (A) OFF and (B) ON. The percentage of aerosols within the region of interest (red box) placed beyond the portable device is 17.7% and 2.2% when the portable device was OFF and ON, respectively. The image is viewed from an oblique angle behind the portable device.

**Figure 4:**
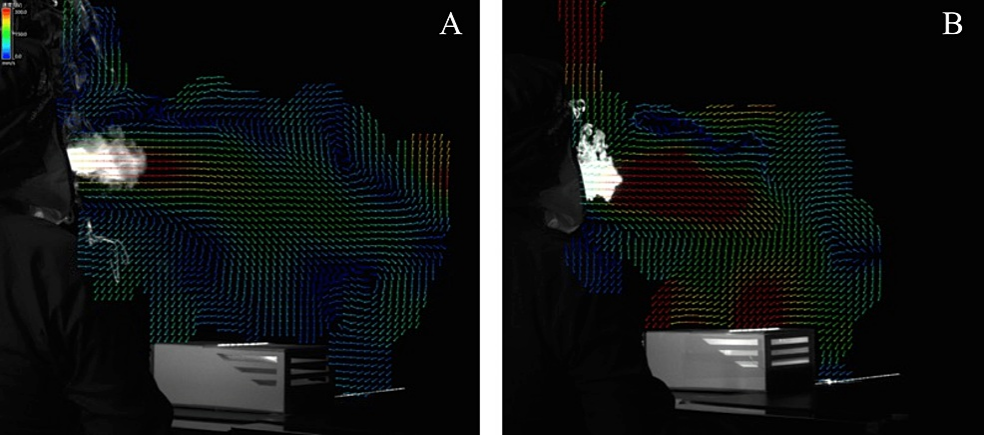
Mean value of the displacement vector with exhaled aerosols from a person when the portable device was (A) OFF and (B) ON. The portable device created airflow toward the inlet ports.

The percentage of aerosols in the ROI was 17.7% and 2.2% when the portable device was OFF and ON, respectively, indicating an 87.6% reduction when the device was used (Figure 3). The mean value of the aerosol displacement vector is also shown in Figure 4. The use of the portable device generated airflow toward the inlet ports and suctioned the suspended aerosols in the air. There was no diffusion of the airflow on the two-dimensional plane irradiated by the PIV laser.

Suction test of the droplets

**Figure 5:**
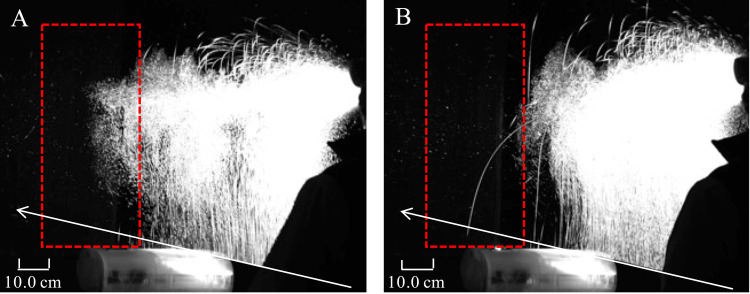
Analysis of the percentage of dispersed droplets when the portable device was (A) OFF and (B) ON. The percentage of droplets within the region of interest (red box) placed beyond the portable device is 13.4% and 1.1% when the portable device was (A) OFF and (B) ON, respectively. The image is viewed from an oblique angle behind the portable device.

**Figure 6:**
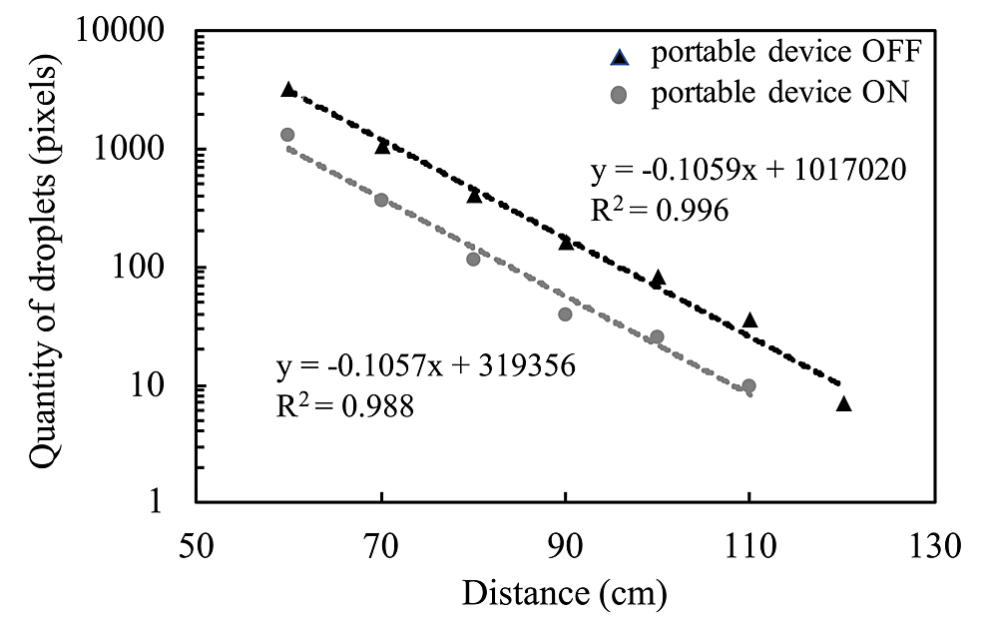
Number of deposited droplets on water-sensitive paper beyond the portable device.

The percentage of droplets in the ROI was 13.4% and 1.1% when the portable device was OFF and ON, respectively, indicating a 91.8% reduction when the device was used (Figure 5). The number of droplets larger than 50 µm that were deposited on the water-sensitive paper beyond the portable device is shown in Figure 6. With the portable device both OFF and ON, 95% of the droplets deposited on the water-sensitive paper deposited up to 90 cm away from the spray point. The number of droplets that splashed more than 100 cm and deposited on the water-sensitive paper was 86 pixels when the portable device was OFF and 26 pixels when the portable device was ON, indicating a 68.7% reduction in droplets deposited using the portable device. Therefore, the number and distance of droplets deposited on the table beyond the portable device decreased. The slopes of the regression line when the portable device was OFF and ON were comparable. Beyond the portable device, droplets were falling naturally, without the influence of the airflow toward the inlet ports.

Verification of the virus removal performance

**Figure 7:**
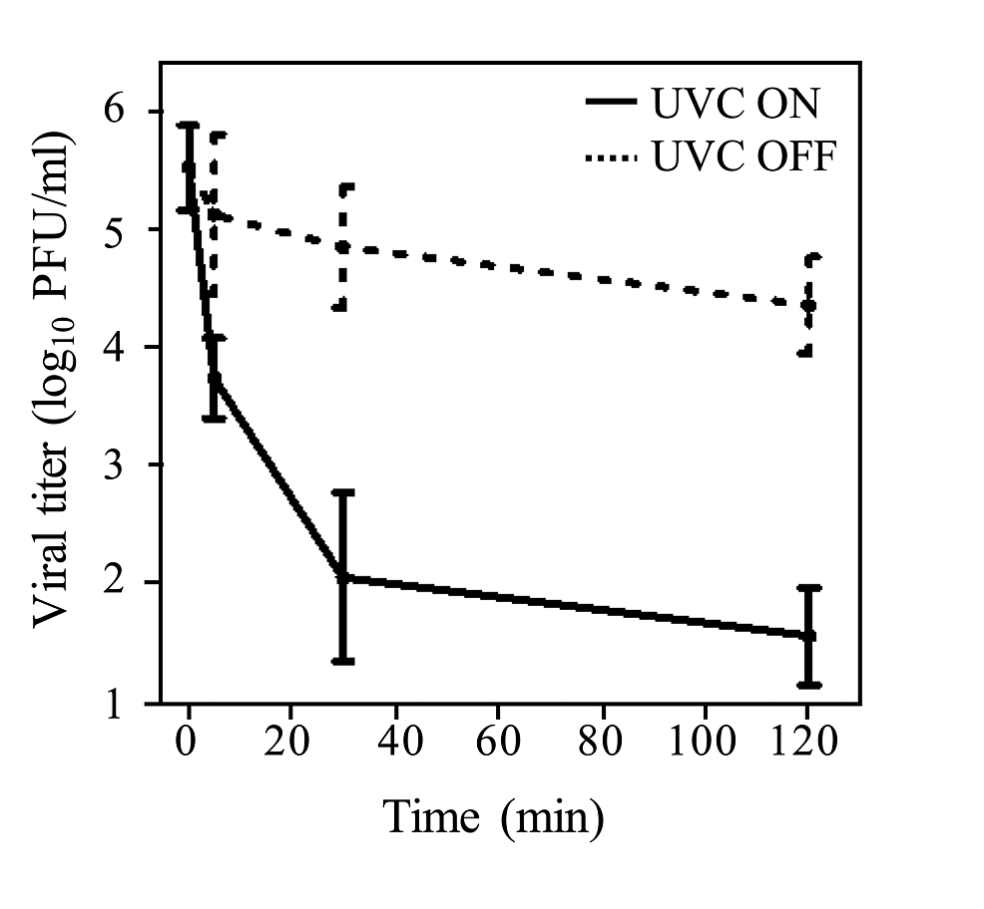
Viral titer of the filter surface when the ultraviolet-C (UVC) is ON and OFF. The viral titer with UVC irradiation was reduced by 99% after 5 minutes and to the lower limit of detection after 2 hours.

On the filter surface, the viral titer with UVC irradiation was reduced by 99% after 5 minutes and to the lower limit of detection after 2 hours (Figure 7). The viral titer without UVC irradiation was reduced by approximately 10% 2 hours after the first dose (Figure 7). On the other side of the filter, the viral titer was not detected with or without UVC irradiation.

## Discussion

We developed a portable device for an indoor environment where people are facing each other and visually demonstrated the descending airflow toward the inlet ports and droplets being suctioned into the portable device. Although the conventional method of suctioning droplets is to place the inlet port close to the droplet generating area [9], our portable device can reduce the number of droplets by 91.8%, even when the device is 50 cm away from the droplet initiation point.

Although the particle size of the splashed droplets with our mist sprayer is unknown, 95% of the droplets deposited up to 90 cm away from the spray point. A previous study [11] showed that most droplets are deposited at a distance of 50-60 cm when using a sprayer that simulates a cough with over 90% droplet deposition at 90 cm from the spray point, similar to our mist sprayer. In this study, the portable device was placed 50 cm away from the spray point, which suggests that the droplets were suctioned by placing the portable device at the distance with the most deposition. The slope of the regression line was −0.1059 when the portable device was OFF and −0.1057 when the portable device was ON, indicating that the droplets fell naturally after passing through the portable device. Microdroplets that are not detected by the water-sensitive paper below 50 μm of particle size might be sucked into the portable device like aerosols, as shown in Figure 3. The number of droplets dispersed more than 100 cm and deposited on the water-sensitive paper was reduced by 68.7% when the portable device was used. In general, by placing the inlet port close to the droplets, the droplets could be efficiently suctioned [12]. Therefore, it is suggested that the droplets that passed near the inlet ports were suctioned, while droplets that passed far from the inlet ports were fallen on the back of the portable device.

In an enclosed room, the aerosol is diffused by the influence of airflow [13]. However, in this study, the airflow toward the inlet ports did not diffuse the aerosol (Figure 3). Our portable device, which inhales aerosol from the top, reduced 87.6% of the aerosol, whereas commercial air purifiers, which exhaust clean air from the top, reduced 60% to 80% of the aerosol [7]. This trend was consistent with the result of a computational fluid dynamics simulation [14]. Therefore, our portable device with inlet ports on its top proved effective prevention of both aerosol and droplet dispersal.

Our portable device is suitable for situations where people cannot be expected to wear a mask such as pediatrics, eating, and acting job. Our portable device is compact and easily movable, and the layout in this experiment can be easily reproduced in an actual situation. One limitation of this study is that we visualized droplets and aerosols in a sagittal plane along the optical path of the PIV laser, and our results did not consider the lateral dispersal of droplets and aerosols. Thus, clean air from the sides of the portable device may cause droplets and aerosols to diffuse laterally. Our portable device is designed as a low-height box type for installation on a table. The portable device can be placed inside the table, exhausting clean air from under the table. Future work will include making the portable device more compact for implementation. Another limitation was that the human influenza virus was used as the model pathogen in this study. It would be informative to conduct experiments using the severe acute respiratory syndrome coronavirus 2 (SARS-CoV-2). We were unable to use SARS-CoV-2 in this study because of institutional restrictions. We intend to establish a safer experimental system and evaluate the direct effects of the device on SARS-CoV-2.

## Conclusions

Our novel portable device has unique features such as good suction and falling the dispersed droplets in the indoor environment where people are facing each other. In the same situation, the device also has sufficient power to suck up and remove suspended aerosols contained in exhaled air. With the combination of the HEPA filter and UVC, the device captures and sanitizes any virus present on the filter surface, ensuring no active virus is detected on the exhaust side.
